# Simultaneous enhancement of cellular and humoral immunity by the high salt formulation of Al(OH)_3_ adjuvant

**DOI:** 10.1038/cr.2017.14

**Published:** 2017-01-20

**Authors:** Min Luo, Bin Shao, Jia-yun Yu, Ting Liu, Xiao Liang, Lian Lu, Ting-hong Ye, Zhi-yao He, Heng-yi Xiao, Xia-wei Wei

**Affiliations:** 1 Lab of Aging Research and Nanotoxicology, State Key Laboratory of Biotherapy, West China Hospital, Sichuan University, Chengdu, Sichuan 610041, China

## Dear Editor,

Adjuvants play essential roles in vaccination by enhancing and/or shaping antigen-specific immune responses^1^. They are commonly used in every type of vaccines, e.g., cancer vaccines, which have been proved to be a promising approach for cancer immune therapy^2^. Aluminum-based adjuvants have been widely used in human vaccination for decades^3^. Despite the robust induction of antibody-mediated immune responses, a limitation of aluminum-based adjuvants is their weak stimulation of cell-mediated immunity^4^. Thus, the development of safe, stable and effective adjuvants with the ability to enhance both humoral and cellular immune responses during clinical vaccination remains challenging. As the major antigen-presenting cells (APCs) to initiate immune responses, dendritic cells (DCs) can be triggered by environmental stimulation to elicit different phenotypes and functions^5,6^. Moreover, cross-presentation of extracellular protein antigens by DCs contributes to the generation of cytotoxic T lymphocyte (CTL) responses^7^. Sodium chloride (NaCl) is closely related to our daily lives and has recently been found to have potential effects on the development of autoimmune diseases^8,9^ and specific states of macrophages^10^. However, the impacts of high concentrations of salt on the functions of DCs and the immune system have not been studied. The use of high salt formulation in combination with aluminum adjuvants in protein vaccines remains to be explored.

**Figure 1:**
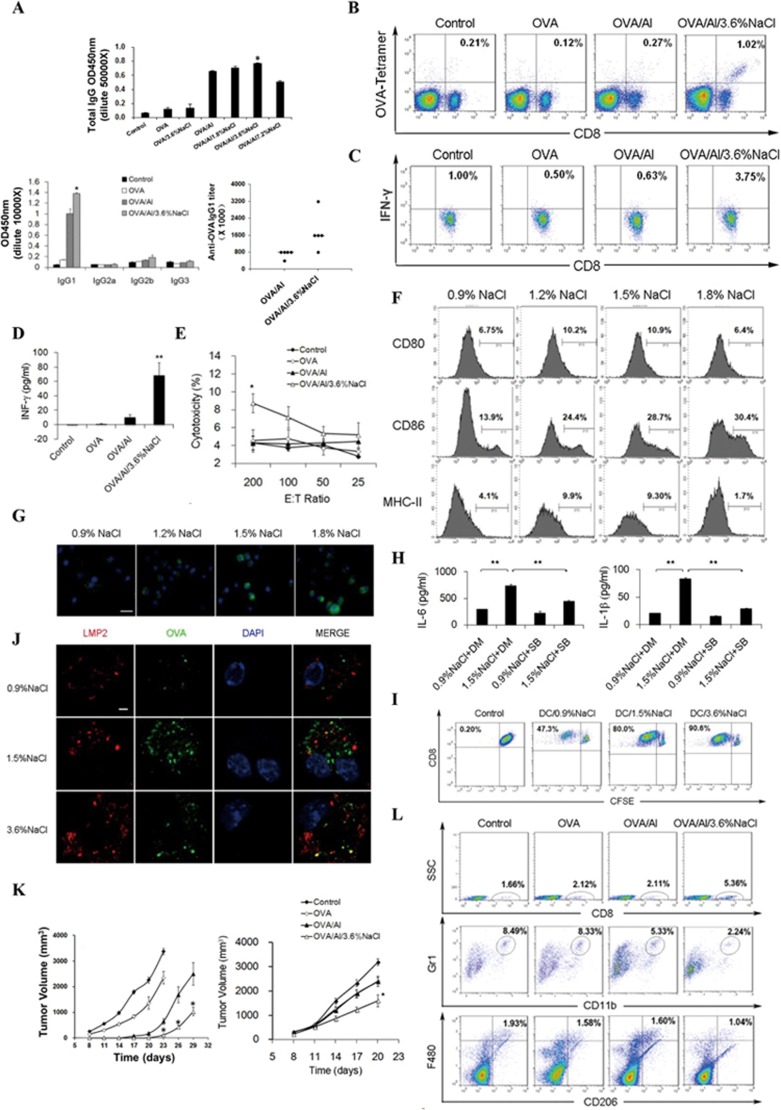
Simultaneous enhancement of cellular and humoral immunity by the high-salt formulation of Al(OH)_3_ adjuvant. **(A)** High salt concentration enhances Al(OH)_3_ adjuvant-induced humoral immunity. C57BL/6 mice (*n* = 5 per group) were vaccinated subcutaneously three times with OVA/Al complex containing different concentrations of NaCl (5 μg OVA per mouse). Seven days after the third immunization, the mouse serum was collected and levels of the total IgG and IgG subclasses and IgG1 titer were determined by ELISA. **(B**-**E)** OVA/Al/high salt vaccine induces specific cellular immunity. C57BL/6 mice were immunized in the same way as described in **A**. Lymphocytes were isolated from the spleen and further incubated *in vitro* with CD8^+^-specific OVA_257–264_ peptides (10 μg/ml) for 3 days. The generation of CD8^+^ CTLs was determined by FCM using PE-conjugated H-2K^b^/OVA_257–264_tetramer **(B)**. The expression of IFN-γ was examined by FCM **(C)** and ELISA **(D)**. The same lymphocytes were tested for CTL-mediated cytotoxicity against E.G7-OVA cells by a standard 6 h ^51^Cr release assay **(E)**. **(F)** High concentrations of NaCl promote the maturation of DCs *in vitro*. Bone marrow-derived DCs isolated from C57BL/6 mice were cultured in medium with the indicated NaCl concentrations for 48 h and the expression of maturation markers was analyzed by FCM. **(G)** High concentrations of NaCl promote the antigen uptake of DCs *in vitro*. DCs were incubated with 2 μg/ml Alexa Fluor 488-labeled OVA for 1 h at 37 °C in medium with the indicated NaCl concentrations and analyzed under a fluorescent microscope. Scale bars, 20 μm. **(H)** High concentration of NaCl promotes secretion of the pro-inflammatory cytokines by DCs through the p38 MAPK pathway *in vitro*. DCs were pretreated with 10 μM SB203580 (SB) or DMSO (DM) for 2 h and then treated with 0.9% or 1.5% NaCl for 48 h in the presence of 5 μM SB or DM. The levels of pro-inflammatory cytokines in the supernatant were measured by ELISA. **(I**-**J)** High salt concentration induces antigen cross-presentation in DCs. DCs were cultured in medium with the indicated NaCl concentrations containing 10 μg/ml OVA. Then the stimulated DCs were co-cultured with CFSE-labeled CD8^+^ T cells from OT-I mice. The proliferation of T cells was assessed by FCM after 3 days of co-culture **(I)**. DCs were treated with 2 μg/ml Alexa Fluor 488-labeled OVA in the presence of the indicated NaCl concentrations for 1 h, stained with rabbit anti-LMP2/Cy3 antibody and DAPI, and visualized under a confocal laser scanning microscope. Scale bars, 5 μm **(J)**. **(K)** High-salt formulation potentiates the antitumor effect of OVA/Al vaccine *in vivo*. In a prophylactic model (left), C57BL/6 mice (*n* = 10 per group) were immunized with different vaccines for three times and then challenged subcutaneously with 3 × 10^6^ E.G7-OVA cells 1 week after the third immunization. In a therapeutic model (right), mice (*n* = 7 per group) were treated by subcutaneous injection of different vaccines once a week for 3 weeks starting on day 3 after subcutaneously introduction of 3 × 10^6^ E.G7-OVA cells. The tumor volume was measured every 3 days. **(L)** The high-salt formulation of OVA/Al diminished immune suppressive cells in tumor microenvironment. After the third treatment, a single-cell suspension of tumor tissues was prepared. The percentages of infiltrating CD8^+^ lymphocytes, MDSCs and M2 macrophages were analyzed by FCM.

Here we developed a new formulation of aluminum hydroxide (Al(OH)_3_) adjuvant with a high salt concentration and utilized the OVA model antigen and the HBsAg antigen to evaluate its adjuvant effect. The serum ELISA results showed that the increase of the total IgG level reached a peak at 3.6% NaCl (Figure 1A). Compared with the adjuvant prepared in regular NaCl concentration (0.9%), the high-salt formulation of Al(OH)_3_ mildly improved OVA-induced production of IgG1-associated Th2 responses rather than IgG2a- and IgG2b-associated Th1 responses (Figure 1A). Similar results were also observed in the HBsAg model (Supplementary information, Figure S1A). These data suggest that the high-salt formulation of Al(OH)_3_ enhances humoral immunity by stimulating the Th2 response.

Next, we assessed whether the new formulation can enhance the induction of cell-mediated immunity. We found that vaccination of mice with OVA/Al/3.6% NaCl dramatically increased the frequency of OVA-specific CD8^+^ T cells as detected by PE-conjugated OVA_257–264_-bound H-2k^b^ tetramer compared with vaccination with OVA/Al (Figure 1B). In addition, vaccination with OVA/Al/3.6% NaCl not only dramatically increased the frequency of CD8^+^ INF-γ-producing CTLs, but also increased the secretion of INF-γ (Figure 1C and 1D). Similar results were also obtained in the HBsAg vaccine model (Supplementary information, Figure S1B and S1C). Moreover, T lymphocytes derived from the mice immunized with OVA/Al/3.6% NaCl exhibited higher cytotoxicity against E.G7-OVA cells (a cell line expressing OVA peptides) as revealed by a standard 51Cr-release assay (Figure 1E). Notably, there was no significant increase in the percentages of CD4^+^ INF-γ-producing Th1 cells after high salt treatment in either the OVA or HBsAg vaccine model (Supplementary information, Figure S1D), indicating that high salt helped to enhance the induction of antigen-specific CTLs, bypassing the CD4^+^ T helper arm. Also, there was no increase of the IL-4 level after immunization with OVA/Al/3.6% NaCl compared with the OVA/Al group (Supplementary information, Figure S1E).

We next examined whether and how high salt influences the activation of DCs. Since a NaCl concentration of 1.8% caused increased apoptosis and cell death of DCs (Supplementary information, Figure S1J), our subsequent *in vitro* experiments mainly focused on the concentrations of 1.2% and 1.5%. Interestingly, high salt significantly promoted maturation and antigen uptake ability of DCs *in vitro* (Figure 1F and 1G and Supplementary information, Figure S1F). Moreover, high salt promotes the expression of inflammatory cytokines in DCs at both mRNA and protein levels (Supplementary information, Figure S1G and S1H). As MAPK pathways were reported to have roles in cellular responses to stress stimulation^11^, we explored the possibility of the involvement of the MAPK signaling pathway in DC activation. The phosphorylation of p38 MAPK significantly increased after hours of high salt treatment (Supplementary information, Figure S1I). Furthermore, the increased release of inflammatory cytokines could be abolished by the addition of a p38 inhibitor SB203580 (Figure 1H).

Efficient cross-presentation of extracellular proteins by DCs has an important role in the initiation of immune responses^7^. Here, we used CD8^+^ T cells from OT-I mice (specific for the H-2K^b^/OVA complex) to test whether high salt-stimulated DCs can perform cross-presentation of soluble OVA. The FCM analysis of CFSE fluorescence showed a 2-fold increase in CD8^+^ T-cell proliferation upon co-culture with high salt-treated DCs (Figure 1I). Also, more clusters of DCs and CD8^+^ T cells were observed and enhanced secretion of IL-2 was detected in the supernatants during the co-culture process (Supplementary information, Figure S1K and S1L). Moreover, confocal microscopy revealed that more OVA co-localized with LMP2 (a marker of the immunoproteasome) in high salt-treated DCs, suggesting a more effective processing of antigens (Figure 1J). Collectively, the above data indicate that high salt can significantly enhance cross-presentation of extracellular antigens by DCs, which might play a critical role in the induction of antigen-specific CTL activity.

Next, we examined the *in vivo* antitumor effect of the OVA/Al/high salt vaccine using the E.G7-OVA tumor model in mice. In either the prophylactic or therapeutic vaccine model, immunization of the OVA/Al/high salt complex inhibited tumor growth more effectively compared with the other groups (Figure 1K). The antitumor effect of the OVA/Al/3.6% NaCl vaccine was further studied in an adoptive cellular/serum therapy model. Lymphocytes from mice immunized with OVA/Al/3.6% NaCl effectively inhibited tumor growth, while the serum showed no apparent antitumor effect (Supplementary information, Figure S1M and S1N). To investigate the function of immune cell subsets in the antitumor activity elicited by the high salt formulation, we depleted CD4^+^/CD8^+^ T cells or NK cells through the injection of monoclonal antibodies. Only depletion of CD8^+^ T lymphocytes and NK cells showed effective abrogation of the antitumor effect induced by the OVA/Al/3.6% NaCl vaccine (Supplementary information, Figure S1O). Taken together, these results suggest that a high-salt formulation of OVA/Al vaccine exhibited an enhanced antitumor effect *in vivo* through CD8^+^ CTL-mediated cellular immunity independent of CD4^+^ T cells.

To further study the role of high-salt formulation in the strong antitumor effect, we assessed the changes in immune cell subsets after the immunization. Both central memory and effector memory T cells in CD4^+^ and CD8^+^ T cell populations significantly increased in the OVA/Al/high salt group (Supplementary information, Figure S1P). We also observed an increase in percentages of splenic CD4^+^ and CD8^+^ T cells in the OVA/Al/high salt group (Supplementary information, Figure S1P). As an immune suppressive tumor microenvironment might be a major determinant of the poor outcome of tumor immunotherapy^12^, we further investigated whether the high-salt vaccine alters the immune microenvironment in tumors. We found that tumor-infiltrating lymphocytes increased, whereas myeloid-derived suppressor cells (MDSCs) and M2 macrophages decreased significantly in OVA/Al/3.6% NaCl-treated mice (Figure 1L). These observations indicate that high salt-containing vaccine may alter the tumor microenvironment to favor more potent antitumor effects.

For safety assessment, no significant differences were observed between normal mice and the mice immunized with aluminum and high salt, as determined by appearance, body weight, fecal and urinary excretion, and H&E staining of the vital organs. All the mice were under good physiological conditions as shown by the monitoring of heart rate and mean arterial pressure, even when the NaCl concentration reached as high as 14.4% (16 times higher than the normal osmolality; Supplementary information, Table S1).

In conclusion, we discovered a novel and safe formulation (high salt formulation) of Al(OH)_3_ adjuvant, which, aside from maintaining the induction of humoral immune response by normal Al-adjuvant, could further enhance the cellular immune response; and this effect may be closely related to the activation of and antigen cross-presentation by DCs. Consequently, the OVA/Al/high salt formulation exhibited a significant antitumor effect against the E.G7-OVA tumor model *in vivo*, which might be largely due to CD8^+^ CTL-mediated cellular immunity and independent of CD4^+^ T cells. The most interesting and surprising finding of this study is that the adjuvant effects could be significantly improved simply by altering the NaCl concentration in vaccines and such formulation is safe, easy to prepare, and of low cost. This concept may assist in the design of broad vaccine formulations and the development of safer and more effective adjuvants for therapeutic uses.

## Supplementary Information

Supplementary information, Figure S1 Related to Figure 1. (A) High salt concentration enhances Al(OH)_3_ adjuvant-induced humoral immunity in mice.

Supplementary information, Table S1 Cardiovascular (heart rate & blood pressure) parameters following high salt/Al vaccination
