# Supplementary material for: Simultaneous enhancement of cellular and humoral immunity by the high salt formulation of Al(OH)3 adjuvant
Source: Cell Res. 2017 Jan 20;27(4):586–9. doi: 10.1038/cr.2017.14 (PMC5385609; doi:10.1038/cr.2017.14)
Supplement: Supplementary information, Figure S1 — Related to Figure 1. (A) High salt concentration enhances Al(OH)3 adjuvant-induced humoral immunity in mice. [file cr201714x1.pdf]

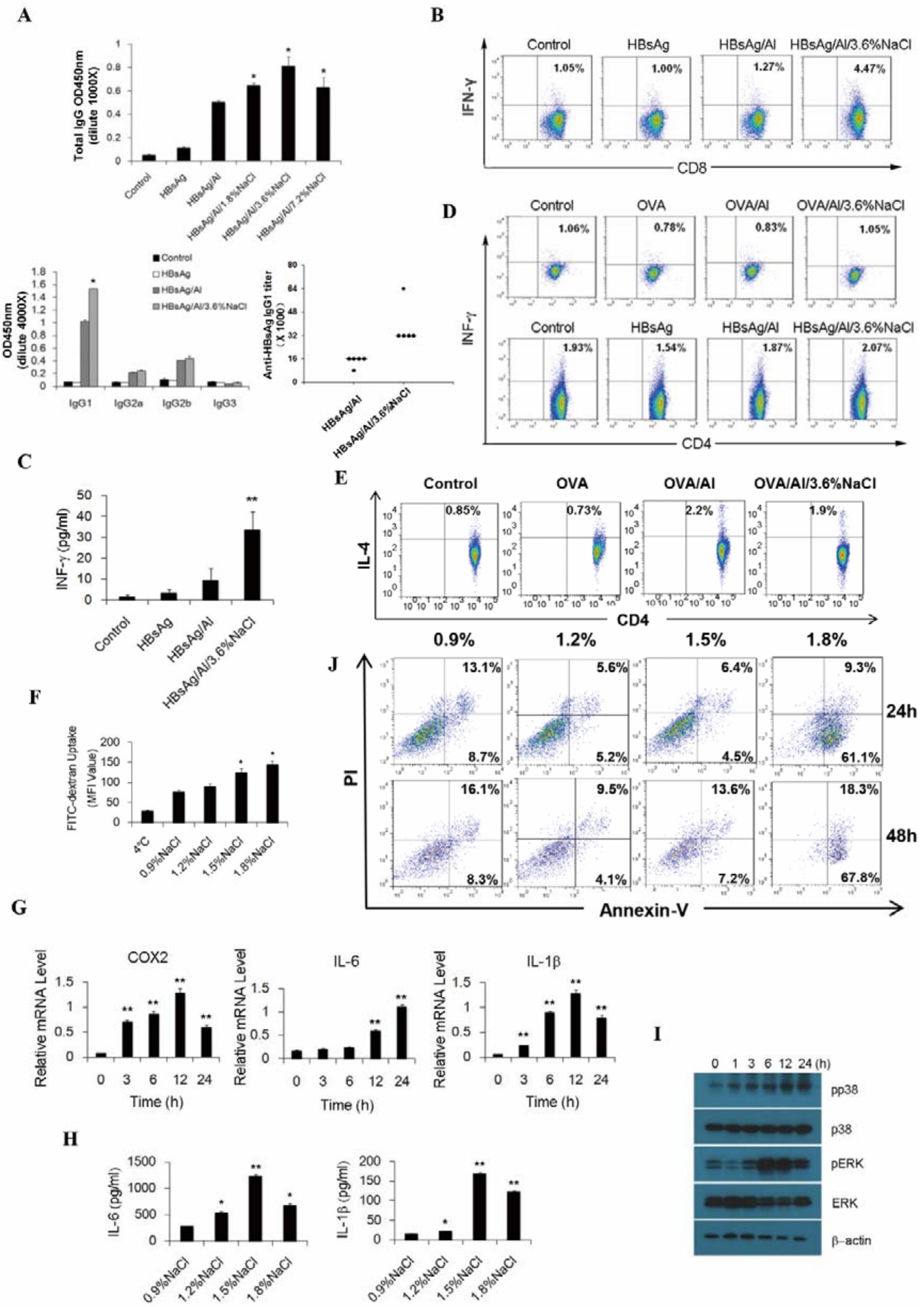
A
Total IgG OD450nm (dilute 1000X)
Control
HBsAg
HBsAg/Al
HBsAg/Al/1.8%NaCl
HBsAg/Al/3.6%NaCl
HBsAg/Al/7.2%NaCl
OD450nm (dilute 4000X)
IgG1
IgG2a
IgG2b
IgG3
Anti-HBsAg IgG1 titer (X 1000)
HBsAg/Al/3.6%NaCl
B
Control
HBsAg
HBsAg/Al
HBsAg/Al/3.6%NaCl
1.05%
1.00%
1.27%
4.47%
IFN-γ
CD8
D
Control
OVA
OVA/Al
OVA/Al/3.6%NaCl
1.06%
0.78%
0.83%
1.05%
1.93%
1.54%
1.87%
2.07%
INF-γ
CD4
C
INF-γ (pg/ml)
E
0.85%
0.73%
2.2%
1.9%
IL-4
F
FITC-dextran Uptake (MFI Value)
4°C
0.9%NaCl
1.2%NaCl
1.5%NaCl
1.8%NaCl
J
0.9%
1.2%
1.5%
1.8%
13.1%
5.6%
6.4%
9.3%
8.7%
5.2%
4.5%
61.1%
24h
16.1%
9.5%
13.6%
18.3%
8.3%
4.1%
7.2%
67.8%
48h
PI
Annexin-V
G
COX2
IL-6
IL-1β
Relative mRNA Level
Time (h)
H
IL-6 (pg/ml)
IL-1β (pg/ml)
I
0 1 3 6 12 24 (h)
pp38
p38
pERK
ERK
β-actin

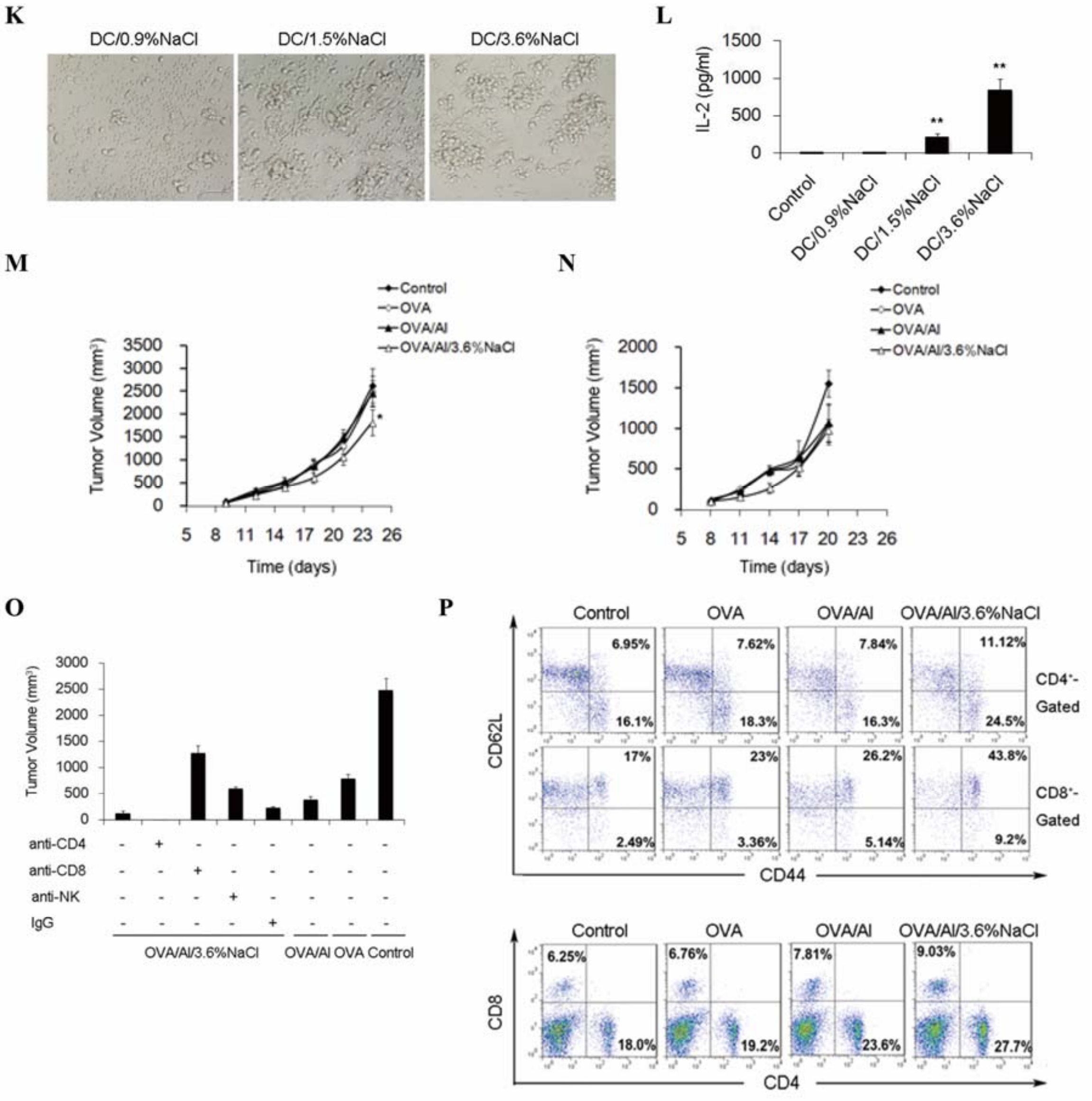
K
DC/0.9%NaCl
DC/1.5%NaCl
DC/3.6%NaCl
L
IL-2 (pg/ml)
Control
DC/0.9%NaCl
DC/1.5%NaCl
DC/3.6%NaCl
M
Control
OVA
OVA/Al
OVA/Al/3.6%NaCl
Tumor Volume (mm³)
Time (days)
N
Control
OVA
OVA/Al
OVA/Al/3.6%NaCl
Tumor Volume (mm³)
Time (days)
O
Tumor Volume (mm³)
anti-CD4
anti-CD8
anti-NK
IgG
OVA/Al/3.6%NaCl
OVA/Al
OVA
Control
P
Control
OVA
OVA/Al
OVA/Al/3.6%NaCl
CD62L
CD44
CD4⁺-Gated
CD8⁺-Gated
6.95%
16.1%
7.62%
18.3%
7.84%
16.3%
11.12%
24.5%
17%
2.49%
23%
3.36%
26.2%
5.14%
43.8%
9.2%
CD8
CD4
6.25%
18.0%
6.76%
19.2%
7.81%
23.6%
9.03%
27.7%

Q

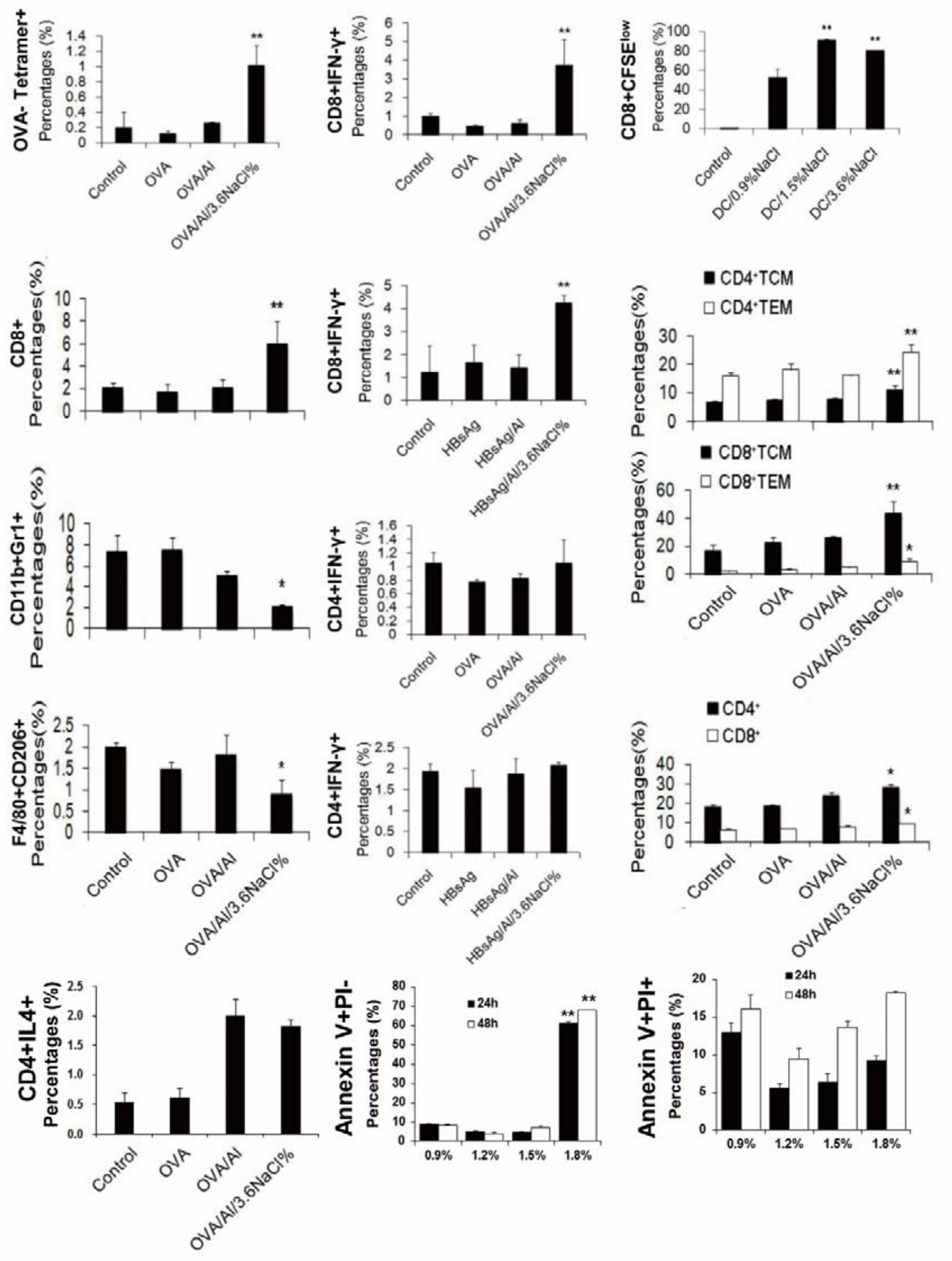

Supplementary information, Figure S1, Related to Figure 1.

(A) High salt concentration enhances $Al(OH)_3$ adjuvant-induced humoral immunity in mice. BALB/c mice (n = 5 per group) were vaccinated s.c. 3 times with HBsAg/Al complex containing different concentrations of NaCl (1μg HBsAg per mouse). 7 days after the 3rd

immunization, serum was collected and the specific total IgG, IgG subclasses and IgG1 titer were determined by ELISA. **(B-C)** OVA/Al/high salt vaccine induces specific cellular immunity. BALB/c mice were immunized in the same way as described in **A**. Lymphocytes were isolated from spleen and further incubated *in vitro* with HBsAg (10 μg/ml) for 3 days. The expression of IFN-γ was examined by FCM **(B)** and ELISA**(C)**. **(D)**Antigen/Al/high salt vaccine has no effect on $CD4^+$ Th1 cells. Lymphocytes were stimulated with $CD4^+$ specific $OVA_{323-339}$ peptide or HBsAg for 3 days and cytoplasmic expression of IFN-γ in $CD4^+$ T cells was examined by FCM both in OVA and HBsAg model. **(E)**High concentration of NaCl has no effect on the IL-4 secretion induced by Al. Lymphocytes were stimulated with $CD4^+$ specific $OVA_{323-339}$ peptide for 3 days and cytoplasmic expression of IL-4 in $CD4^+$ T cells was examined by FCM in OVA model.**(F)**High concentration of NaCl promotes the antigen uptake of DCs *in vitro*. DCs were incubated with FITC-dextran for 1h at 37°C in the medium of indicated NaCl; the mean fluorescence intensity (MFI) of $CD11c^+$ cells was analyzed by FCM.**(G-H)**High concentration of NaCl promotes the expression of pro-inflammatory cytokines secretion of DCs either in mRNA or in protein level. DCs were treated in medium containing indicated concentration of NaCl for indicated time. mRNA was extracted and analyzed by quantitative RT-PCR **(G)** and protein in the supernatant was measured by ELISA **(H)**. **(I)** High concentration of NaCl activated the MAPK signaling pathways in DCs. DCs were treated as in **G** in 1.5% NaCl, cell lysates were analyzed by Western Blots.**(J)**A moderate increase of NaCl concentration didn't affect the viability of DCs. DCs were cultured in medium with indicated concentration of NaCl for 24h and 48h;the apoptosis and viability of $CD11c^+$ DCs were examined by Annexin V and PI staining.**(K-L)** High salt concentration induces the antigen cross-presentation in DCs. DCs were cultured in medium with indicated NaCl containing 10 μg/ml OVA for different time. Then stimulated DCs were co-cultured with $CD8^+$T cells from OT-I mice. Typical images of clusters of DCs and $CD8^+$ T cells after 24h of co-culture. Scale bars, 50 μm (**K**). The IL-2 secretion in the culture supernatants was

measured by ELISA after 72h(**L**). **(M-O)** High salt formulation of OVA/Al vaccine exhibited an enhanced antitumor effect *in vivo* through the $CD8^+$ CTLs-mediated cellular immunity independent of the help from $CD4^+$ cells. Lymphocytes from vaccinated mice (10^7/mouse) were injected i.v. into recipient mice 1 day before, 1day and 3 day after tumor cell inoculation(*n* = 7 per group)**(M)**; Tumor-bearing mice (*n* = 7 per group) were injected i.v. with 250 μl of the serum from vaccinated mice twice per week for 3 weeks in the serum adoptive therapy**(N)**; Mice (*n* = 7 per group) were injected i.p. with 500 μg of indicated mAb 1 day before the first immunization, and then administered 150 μg twice a week for 3 weeks. 3×10^6 E.G7-OVA tumor cells were inoculated in each group after the 3rd immunization. Tumor volumes presented were recorded on Day 23 after tumor cell injection **(O)**. **(P)**High salt formulation of OVA/Al alters the T cell phenotypes. The percentages of memory T cells in the spleen of the mice in the prophylactic model or T cells in spleens from the mice in the therapeutic model were detected by FCM.**(Q)**All the quantitative data of FCM results are presented as mean ± s.d., $n = 3$, * $p < 0.05$, ** $p < 0.01$ compared to $Al(OH)_3$ group by one-way ANOVA test. The representative flow plot figures from three independent experiments are presented.
