# Supplementary material for: Simultaneous enhancement of cellular and humoral immunity by the high salt formulation of Al(OH)3 adjuvant
Source: Cell Res. 2017 Jan 20;27(4):586–9. doi: 10.1038/cr.2017.14 (PMC5385609; doi:10.1038/cr.2017.14)
Supplement: Supplementary information, Table S1 — Cardiovascular (heart rate & blood pressure) parameters following high salt/Al vaccination [file cr201714x2.pdf]

Supplementary information, Table S1 Cardiovascular (heart rate & blood pressure) parameters following high salt/Al vaccination

	Time point	Group				
		Control	OVA/Al	OVA/Al/3.6%NaCl	OVA/Al/7.2%NaCl	OVA/Al/14.4%NaCl
HR (Per min)	6h post-vaccine I	624.0±57.2	588.6±61.4	570.1±37.0	616.0±64.3	563.0±31.5
	3d post-vaccine I	651.0±57.9	608.6±50.1	602.2±13.2	615.0±10.4	658.3±46.8
	7d post-vaccine I	662.2±44.1	648.3±64.0	640.1±30.2	646.8±11.0	696.7±32.1
	3d post-vaccine II	674.1±40.7	671.7±64.6	640.7±68.0	643.9±65.5	663.4±54.1
	7d post-vaccine II	658.1±63.4	632.7±54.6	622.9±30.2	625.3±54.9	655.2±70.0
	3d post-vaccine III	646.2±46.6	671.0±35.2	625.5±22.3	661.2±58.6	655.4±52.8
	7d post-vaccine III	653.1±52.3	668.8±49.9	637.6±67.9	637.9±45.0	649.8±43.7
MAP (mm Hg)	6h post-vaccine I	93.5±2.6	73.2±8.4	76.8±9.7	85.1±4.1	79.3±6.1
	3d post-vaccine I	80.7±1.4	76.8±1.6	77.1±4.0	78.7±8.2	79.2±2.0
	7d post-vaccine I	85.2±7.5	88.4±7.1	84±7.5	87.9±7.9	85.8±3.5
	3d post-vaccine II	89.2±2.0	87.4±2.4	93.4±8.2	85.7±7.7	84.7±3.3
	7d post-vaccine II	90.3±6.4	85.9±8.9	87.2±3.4	88.2±7.1	87.4±6.5
	3d post-vaccine III	85.6±2.6	83.7±3.9	91.2±4.2	87.4±6.8	83.7±3.8
	7d post-vaccine III	88.7±5.9	86.6±7.1	89.4±5.3	88.3±7.8	86.6±5.9

C57BL/6 mice (n = 3 per group) were vaccinated s.c. 3 times with OVA/Al complex containing different concentrations of NaCl as indicated. We monitored the heart rate (HR) and mean arterial pressure (MAP) of mice at early and late timepoints after vaccination by the tail-cuff method (BP-98A). Values represent mean ± s.d. No significant differences were observed between the different vaccination groups.
